# Urinary lipid metabolites and progression of kidney disease in individuals with type 2 diabetes

**DOI:** 10.3389/fendo.2025.1650498

**Published:** 2025-11-19

**Authors:** Yu Xiao, Caifeng Shi, Songyan Qin, Aiqin He, Xiaomei Wu, Chunsun Dai, Yang Zhou

**Affiliations:** Center for Kidney Disease, The Second Affiliated Hospital of Nanjing Medical University, Nanjing, China

**Keywords:** diabetic kidney disease, lysophosphatidylcholine, phosphatidylcholine, sphingomyelin, fast decline, targeted lipidomics

## Abstract

**Objective:**

A substantial proportion of individuals with type 2 diabetes (T2D) experience a fast decline (FD) in kidney function, a high-risk phenotype not reliably identified by current clinical markers. This study aimed to evaluate the potential of urinary lipid metabolites as novel predictors for the rapid progression of diabetic kidney disease (DKD).

**Methods:**

This investigation employed a dual-phase design comprising cross-sectional screening and longitudinal validation. In the initial phase, targeted lipidomic profiling of urine samples from 152 patients with T2D and DKD and 152 age- and sex-matched individuals with uncomplicated diabetes revealed distinct metabolite patterns. The subsequent validation phase utilized an independent cohort of 248 T2D patients, in which rapid kidney function decline was defined as the highest quartile of annual estimated glomerular filtration rate (eGFR) reduction. Feature selection was performed using machine learning algorithms (random forest and Boruta) to identify potential biomarkers from the differentially expressed metabolites. The prognostic value of these lipid markers for predicting future renal function decline was assessed against clinical variables using receiver operating characteristic (ROC) analysis.

**Results:**

The analysis of fasting spot urine specimens quantified 104 lipid metabolites out of 508 targeted species, with all concentrations normalized to urinary creatinine. The comparative analysis identified 21 lipid metabolites that were significantly upregulated in the DKD group. Feature selection algorithms isolated nine (Boruta) and eight (random forest) candidate biomarkers from this pool. During a median follow-up period of 33 months (IQR 17–47), 62 participants showing the most rapid eGFR decline were classified as the FD group. These individuals exhibited significantly elevated baseline levels of the identified lipid metabolites. The lipid panel demonstrated superior predictive performance for future kidney function decline compared with traditional clinical predictors, including baseline eGFR, hemoglobin A1c, and albuminuria.

**Conclusions:**

Our findings reveal a strong association between urinary lipid metabolites and DKD progression. Specifically, urinary lipid profiling shows promise as a non-invasive tool to identify T2D patients at a high risk for rapid kidney function decline, outperforming the current clinical standard of albuminuria and eGFR.

## Introduction

Diabetic kidney disease (DKD) affects 20%–40% of adults with diabetes (1–3) and substantially elevates their risk of cardiovascular events and mortality (4). Although recent pharmacological advances have improved glycemic and metabolic control, available treatments still cannot fully prevent progression to end-stage kidney disease (ESKD), which often necessitates renal replacement therapy (5).

Clinically, DKD in type 2 diabetes (T2D) is diagnosed based on the presence of albuminuria or a gradual decline in estimated glomerular filtration rate (eGFR) in the absence of other renal pathologies (6). While DKD typically develops after long-standing diabetes, some patients already show signs of kidney impairment at T2D diagnosis. Unlike in type 1 diabetes, where nephropathy and retinopathy frequently co-occur, retinopathy exhibits only moderate specificity and sensitivity as a surrogate for biopsy-confirmed DKD in T2D (7, 8). Renal biopsy is generally not recommended unless atypical features are present, such as active urinary sediment, rapidly rising albuminuria, nephrotic syndrome, or a rapid decline in eGFR. Moreover, a non-albuminuric phenotype of eGFR loss has become increasingly common in diabetes (9–11).

Notably, a subset of T2D patients experience rapid kidney function decline, categorized as fast decline (FD) (12, 13). Early identification of these high-risk individuals is critical for personalized intervention, yet current clinical markers lack sufficient predictive accuracy.

Dysregulated lipid metabolism is a well-established driver of DKD (13), where specific intracellular lipid species like ceramides and diacylglycerols act as direct mediators of renal cell injury (lipotoxicity) rather than mere disease consequences (14). Most prior lipidomic studies, however, have focused on circulating lipids in plasma, which may not fully reflect pathological processes within the kidney. In contrast, the urinary lipidome, as a direct effluent from the kidney, remains a largely untapped resource for biomarker discovery.

To bridge this gap, we conducted a comprehensive targeted lipidomic study in urine. The novelty of our work is twofold: first, we systematically profiled a wide array of lipid species in a well-phenotyped cohort to identify a cross-sectional signature of DKD; second and more importantly, we leveraged a longitudinal design to rigorously evaluate whether specific urinary lipids at baseline can predict the future risk of rapid kidney function decline, a critical unmet need in clinical practice. Our findings contribute a novel, non-invasive predictive tool and provide new pathophysiological insights into the lipid-centric mechanisms of DKD progression.

## Materials and methods

### Ethics statement

All study procedures were conducted in accordance with the ethical principles of the Declaration of Helsinki. The study protocol was reviewed and approved by the Ethics Committee of Nanjing Medical University (approval no. 2019-KY-097). Written informed consent was obtained from every participant prior to enrollment.

#### Human study 1: a cross-sectional study

We recruited adult participants with T2D from outpatients visiting the Department of Internal Medicine at the Second Affiliated Hospital of Nanjing Medical University. T2D was diagnosed according to the 2018 criterial of American Diabetes Association (15). The cohort included 152 individuals with clinically diagnosed DKD as urinary albumin-to-creatinine (UACR) ≥30 mg/g or estimated glomerular filtration rate (eGFR) <60 mL/min/1.73 m^2^ in the absence of signs or symptoms of other primary causes of kidney damage (6). We further selected 152 subjects with uncomplicated T2D with matched age and sex as a matched cohort. We applied chronic kidney disease epidemiology collaboration equation (CKD-EPI equation, http://www.nkdep.nih.gov) to calculate the eGFR. The exclusion criteria were acute kidney injury, acute inflammatory diseases, malignant neoplasm, systemic diseases, or having received kidney replacement therapy.

#### Human study 2: a longitudinal study

An independent cohort of 248 T2D subjects (163 male and 85 female) was established from the same hospital’s physical examination center. The annual eGFR slope for each participant was determined using the least squares method based on measurements from baseline and at least two subsequent time points per year (16). FD in kidney function was defined as belonging to the highest quartile of eGFR slope distribution (17). Fasting spot urine samples were collected from all participants at baseline and stored at -80 °C for subsequent analysis.

### Clinical and laboratory measurement

All human samples were collected, stored, and measured according to the standard operating protocol of the hospital. Blood pressure (systolic and diastolic), body mass index (BMI), waist-to-hip ratio (WHR), fasting blood glucose levels, fasting serum lipid profiles [total cholesterol (TC), triglyceride (TG), high density lipoprotein cholesterol (HDL-c), and low density lipoprotein cholesterol (HDL-c)], kidney functions tests [serum creatinine (SCr), uric acid (UA)], hemoglobin (Hb), hemoglobin A1c (HbA1c), albumin (Alb), and fast spot urine albumin and creatinine levels were determined and recorded. Hypertension was diagnosed if systolic blood pressure (SBP) ≥140 mmHg or diastolic blood pressure (DBP) ≥90 mmHg or if the patient is on antihypertension medication (18).

### Urine sample collection

We collected fasting spot urine in our study, which is a well-established and practical alternative in large-scale clinical and epidemiological research, effectively balancing rigor with feasibility. Key steps were taken to ensure data quality, namely: (1) all samples were collected under a standardized protocol (fasting state) to minimize pre-analytical variability related to diet and hydration; (2) samples were processed and frozen immediately at -80°C after collection to prevent degradation; and (3) all lipid abundances were normalized to urinary creatinine to correct for differences in urine concentration, which is a critical step for spot urine samples and significantly improves reliability.

### Targeted lipidomics analysis

Quality control and targeted lipidomics were performed by Metabo-Profile Biotechnology (Shanghai) Co., Ltd. (Shanghai, China). Sample preparation and derivation were as follows: an aliquot of 20 μL of each urine sample was mixed with 120 μL of standard solution containing 508 lipid metabolites, focusing on major lipid classes relevant to human metabolism (19). Supernatant of 30 μL was added to a 96-well plate after 13,500*g* centrifugation for 10 min at 4°C. After adding 10 μL of freshly prepared derivative reagents (20) to each well, derivation was carried out for 1 h at 60°C. Subsequently, 400 μL of 50% methanol was added. After 4,000*g* centrifugation for 30 min at 4°C, 135 μL of supernatant was transferred to a new 96-well plate for ultra-performance liquid chromatography/targeted quantification mass spectrometry (UPLC/TQMS) analysis. A Waters ACQUITY ultraperformance LC system coupled with a Waters XEVO TQ-S mass spectrometer with an ESI source controlled by MassLynx 4.1 software (Waters, Milford, MA, USA) was used for all analyses based on the published conditions and parameters (21, 22). Raw data files were ultimately processed by targeted metabolome batch quantification (TMBQ) software (v1.0, HMI, Shenzhen, Guangdong, China). Regarding the actual detection rate, of the 508 targeted lipids, a total of 104 were consistently detected and passed our stringent quality control (QC) filters in the fasting spot urine samples. Our QC criteria included a signal-to-noise ratio >10, a coefficient of variation <15% in the pooled quality control samples, and a detection rate >80% across all samples. Quantification for each metabolite in fast spot urine sample was normalized to urinary creatinine concentrations. According to a standard practice in metabolomics data processing, metabolites with more than 20% missing values (or below the detection limit) across all samples were excluded from the analysis. For the remaining metabolites with sporadic missing values, we imputed these with half of the minimum positive value for that specific metabolite across the dataset.

### Statistical analysis

Continuous data were appropriately presented as mean ± standard deviation (SD) or median and interquartile range (IQR) depending on the distribution of variables determined by Kolmogorov–Smirnov test. Differences between groups in continuous variables were tested by *t*-test, Mann–Whitney *U* test, or Wilcoxon signed-rank test, depending on the normality of data and homogeneity of variance, and chi-squared test was used for class variables. The differential lipid metabolites were obtained using univariate statistical analysis with the threshold value of |log_2_ fold change (FC)| ≥1.5 and *p <*0.05.* A* multivariable linear regression was performed to determine if the differences in the urinary lipid metabolites between the groups remain significant after adjusting for potential confounders. A model was built for each lipid metabolite, including group, diabetes duration, HbA1c, and lipid profiles as independent variables. We conducted multivariate logistic regression analyses to determine if the lipid metabolite signature is an independent predictor of rapid kidney function decline. The model was adjusted for relevant clinical covariables, including age, sex, baseline eGFR, HbA1c, and albuminuria. Random forest (RF) or Boruta analysis was carried out to find out candidate biomarkers from these differential lipid metabolites. Specifically, more reliable potential biomarkers were selected by getting the union/intersection of the differential metabolites from univariate statistics and the top 10 important differential metabolites by RF/Boruta. Spearman correlation analysis was performed to evaluate the association between the lipid metabolites and clinical parameters, and the strength of association was presented with *r*-value. Binary logistic regression analysis was used for statistical modeling, while receiver operating characteristic (ROC) curve was performed to assess the clinical benefit of using individual or total differential lipid metabolites as a predictor of fastest eGFR decline quantile. The areas under the receiver operating characteristic curve (AUC) of the different prediction models were compared using DeLong’s test. The analyses were performed using SPSS 25 (process procedure for SPSS version 3.4) and R software. A *p*-value less than 0.05 was considered significant.

## Results

### Shifted urinary lipid composition in DKD

To investigate the association between urinary lipid metabolites and DKD, we performed targeted lipidomic profiling in a cohort of T2D patients. The study included 152 participants diagnosed with DKD and 152 age- and sex-matched individuals with uncomplicated diabetes. As summarized in **Table 1**, the matching procedure successfully balanced the age and sex distribution between the two groups.

**Table 1:** Clinical characteristics of matched subjects with uncomplicated diabetes and DKD in the cross-sectional study.

Clinical characteristics	Uncomplicated diabetes (*n* = 152)	DKD (*n* = 152)	*p*-value
Male sex (%)	96 (63.16%)	98 (64.47%)	0.811
Age (year)	57.50 (49.25, 61.00)	57.00 (51.00, 62.00)	0.939
Duration of diabetes (month)	47.00 (8.00, 137.50)	96.50 (22.00, 185.75)	0.003
BMI (kg/m^2^)	24.34 (22.24, 26.67)	25.43 (23.62, 27.83)	0.011
WHR	0.93 ± 0.07	0.95 ± 0.06	0.059
SBP (mmHg)	130.00 (121.00, 141.00)	138.00 (124.00, 146.00)	0.007
DBP (mmHg)	81.21 ± 9.08	85.65 ± 11.41	<0.001
HR (per minute)	85.00 (78.00, 93.25)	78.00 (70.00, 84.00)	0.807
Hb (g/L)	145.00 (134.00, 155.50)	141.00 (126.50, 154.00)	0.011
Alb (g/L)	46.40 (42.33, 49.18)	43.40 (38.88, 46.73)	<0.001
FBG (mmol/L)	8.19 (6.83, 10.90)	8.75 (6.80, 11.67)	0.080
HbA1c (%)	8.20 (6.70, 10.00)	8.50 (6.90, 10.13)	<0.001
SCr (μmol/L)	64.45 (54.00, 73.75)	72.45 (57.48, 105.05)	<0.001
UA (μmol/L)	292.00 (245.00, 342.00)	343.00 (290.50, 412.50)	<0.001
TC (mmol/L)	4.47 (3.69, 5.34)	4.75 (3.92, 5.55)	0.052
TG (mmol/L)	1.45 (1.03, 2.17)	1.82 (1.18, 2.81)	0.001
HDL-C (mmol/L)	1.13 (0.96, 1.32)	1.05 (0.90, 1.24)	0.006
LDL-C (mmol/L)	2.99 (2.21, 3.79)	2.89 (2.24, 3.75)	0.999
UACR (mg/g)	8.60 (4.60, 14.42)	168.40 (53.69, 697.21)	<0.001
eGFR (mL/min/1.73^2^)	101.25 (92.59, 107.91)	94.27 (65.13, 107.37)	<0.001

Data are presented as mean ± SD, median (IQR), or *n* (%). The two groups were matched for age and sex.

Alb, albumin; BMI, body mass index; DBP, diastolic blood pressure; eGFR, estimated glomerular filtration rate; FBG, fast blood glucose; Hb, hemoglobin; HbA1c, hemoglobin A1c; HDL-C, high density lipoprotein cholesterol; HR, heart rate; LDL-C, low density lipoprotein cholesterol; SBP, systolic blood pressure; SCr, serum creatinine; TC, total cholesterol; TG, triglyceride; UA, uric acid; UACR, urinary albumin creatinine ratio; WHR, waist–hip ratio.

**Figure 1:**
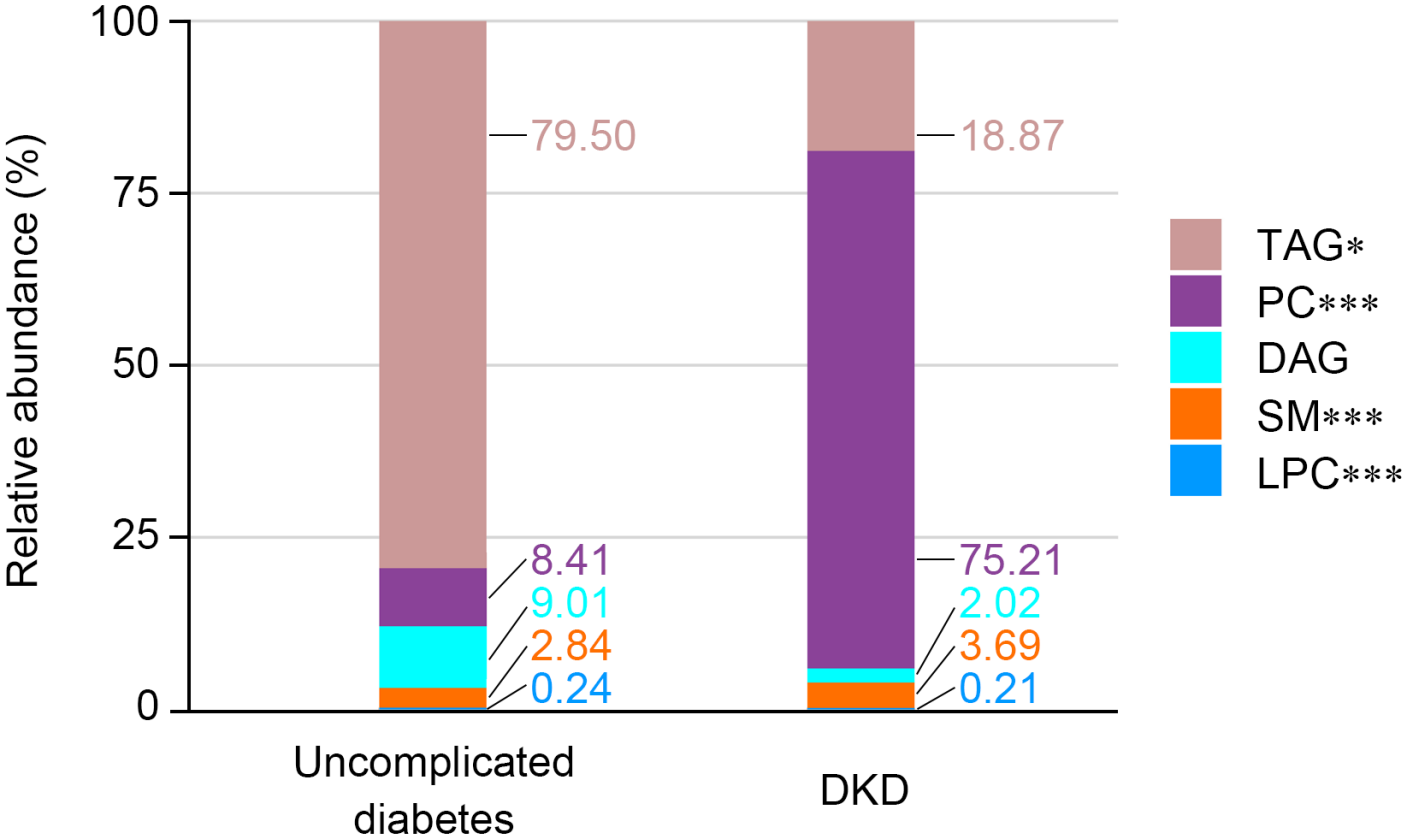
Relative abundance of each metabolite class in spot urine from matched subjects with uncomplicated diabetes and DKD. The stacked bar chart shows the relative abundance of each metabolite class in two groups. Significance levels are marked as *p < 0.05; ***p < 0.001.

Lipidomic profiling of fasting spot urine specimens yielded quantifiable data for 104 lipid metabolites from a predefined set of 508 targets. The abundance of each metabolite is expressed as a ratio to urinary creatinine. Comparative analysis revealed substantial alterations in urinary lipid composition between the groups (**Figure 1**). Specifically, the DKD group exhibited significantly lower relative abundances of triacylglycerol (TAG) and lysophosphatidylcholine (LPC) classes alongside elevated levels of phosphatidylcholine (PC) and sphingomyelin (SM) classes compared with the uncomplicated diabetes group. In contrast, the relative abundance of diacylglycerol (DAG) remained comparable between the two groups.

### DKD is associated with higher urinary concentrations of lipid metabolites

Based on the screening criteria of |log_2_FC| ≥1.5 and *p* < 0.05, we identified 21 lipid metabolites that were significantly altered in the DKD group compared with the matched uncomplicated diabetes group. A comprehensive volcano plot provides a global overview of all detected lipids, highlighting the 21 significantly upregulated metabolites (**Supplementary Figure S1**).

**Figure 2:**
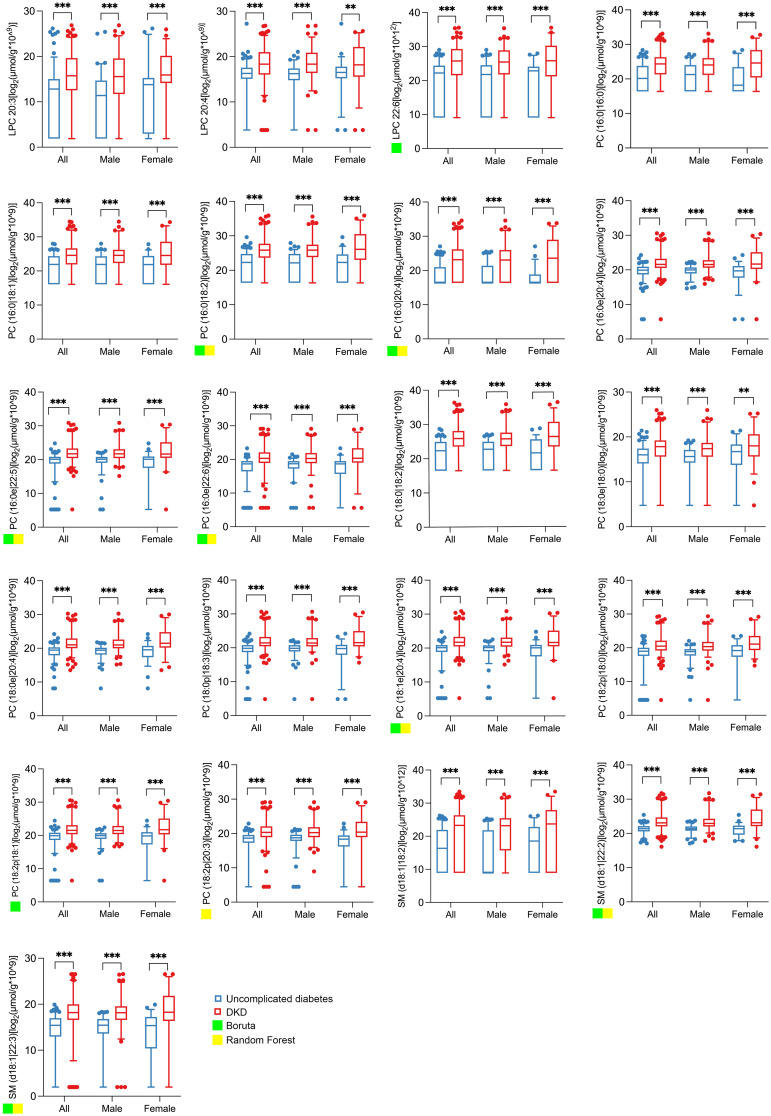
Abundance of the 21 significantly changed lipid metabolites in spot urine from matched subjects with uncomplicated diabetes and DKD. The boxplots show the abundance of 21 qualified differential metabolites selected according to the threshold value of |log_2_FC| ≥ 1.5 and *p* < 0.05 based on univariate statistics. All lipid concentrations were normalized to urinary creatinine to account for variations in urine concentration. The abundance data were log_2_-transformed prior to statistical analysis and visualization to better approximate a normal distribution. Significance levels are marked as **p < 0.01; ***p < 0.001. A green or yellow square indicates that the lipid metabolite is one of the top 10 important differential metabolites selected by Boruta or random forest.

The abundance of the 21 individual metabolites, including LPC 20:3, LPC 20:4, LPC 22:6, PC(16:0|16:0), PC(16:0|18:1), PC(16:0|18:2), PC(16:0|20:4), PC(16:0e|20:4), PC(16:0e|22:5), PC(16:0e|22:6), PC(18:0|18:2), PC(18:0e|18:0), PC(18:0e|20:4), PC(18:0p|18:3), PC(18:1e|20:4), PC(18:2p|18:0), PC(18:2p|18:1), PC(18:2p|20:3), SM(d18:1|18:2), SM(d18:1|22:2), and SM(d18:1|22:3), showed a consistently higher abundance in the DKD group, as visually summarized in **Figure 2**.

In addition, a multivariable linear regression analysis was used to determine if the differences in the 21 urinary lipid metabolites between the DKD and uncomplicated T2D groups remain significant after adjusting for potential confounders. The results confirm that the significant association between the DKD status and the levels of the majority of the 21 lipid metabolites persisted after adjusting for duration of diabetes and HbA1c and lipid profiles (**Supplementary Table S1**).

To identify potential biomarkers from the 21 differential metabolites, we performed feature selection using both RF and Boruta algorithms, retaining metabolites with the top 10 importance scores. The Boruta analysis selected nine metabolites [LPC 22:6, PC(16:0|18:2), PC(16:0|20:4), PC(16:0e|22:5), PC(16:0e|22:6), PC(18:1e|20:4), PC(18:2p|18:1), SM(d18:1|22:2), and SM(d18:1|22:3)], indicated by green squares in **Figure 2**. The RF algorithm selected eight metabolites [PC(16:0|18:2), PC(16:0|20:4), PC(16:0e|22:5), PC(16:0e|22:6), PC(18:1e|20:4), PC(18:2p|20:3), SM (d18:1|22:2), and SM(d18:1|22:3)], marked with yellow squares in **Figure 2**.

### Correlation of urinary lipid metabolites with clinical variables

**Figure 3:**
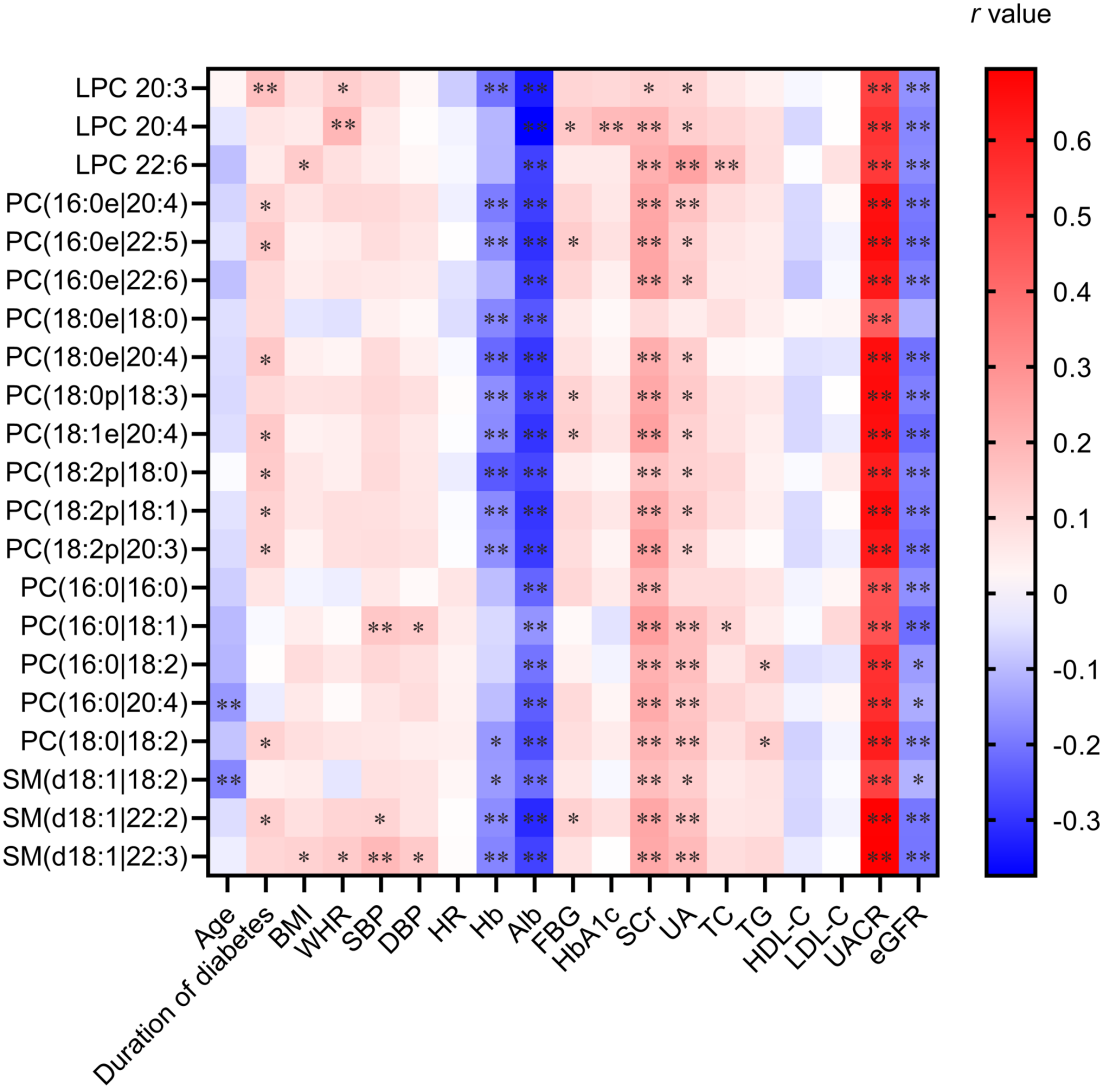
Heatmaps of Spearman correlation coefficients of 21 significantly changed metabolites with representative clinical characteristics. The heatmap visualizes correlation coefficients (*r*) between the 21 metabolites and key clinical variables. Color intensity reflects the strength and direction (red: positive; blue: negative) of the correlation. Significance levels are marked as *p < 0.05 and **p < 0.01.

The correlation analyses consistently demonstrated that all 21 differential lipid metabolites exhibited significant inverse correlations with both eGFR and albumin and positive correlations with both serum creatinine and urinary albumin levels across the overall and matched cohorts (**Figure 3**). This consistent pattern strongly reinforces the association between this distinct urinary lipid profile and the severity of kidney impairment in DKD.

Furthermore, these lipid metabolites showed a high degree of positive co-regulation among themselves (**Supplementary Figure S2**), suggesting that their alterations occur in a synchronized manner rather than in isolation. This coordinated shift implies the dysregulation of specific, interconnected lipid metabolic pathways during the progression of DKD.

### Changes in urinary lipid metabolites and presence of DKD in individuals with T2D

The ROC curve analysis demonstrated that the collective panel of lipid metabolites, whether comprising the nine Boruta-selected, eight RF-selected, or all 21 differential metabolites, achieved identical and superior diagnostic accuracy for DKD (AUC = 0.81). The individual metabolites also showed considerable discriminatory power, with AUCs ranging from 0.68 to 0.80 (**Table 2**). These results substantiate the utility of both specific and combined urinary lipid biomarkers in identifying DKD among patients with T2D.

**Table 2:** ROC analyses of individual and combined lipid metabolites for DKD in the cross-sectional study.

Panel of lipid metabolites	AUC (95% CI)	Sensitivity	Specificity
FC (*n* = 21)	0.81 (0.76, 0.86)	0.71	0.77
FC + Br (*n* = 9)	0.81 (0.76, 0.86)	0.66	0.82
FC + RF (*n* = 8)	0.81 (0.76, 0.86)	0.56	0.93
SM (d18:1|22:3)	0.80 (0.76, 0.85)	0.55	0.93
SM (d18:1|22:2)	0.80 (0.75, 0.85)	0.74	0.78
PC (16:0e|22:5)	0.79 (0.74, 0.84)	0.65	0.88
PC (18:1e|20:4)	0.79 (0.73, 0.84)	0.66	0.88
PC (18:0e|20:4)	0.78 (0.73, 0.83)	0.74	0.73
PC (16:0e|20:4)	0.78 (0.73, 0.83)	0.57	0.91
PC (18:0p|18:3)	0.78 (0.73, 0.83)	0.59	0.87
PC (18:2p|18:1)	0.78 (0.73, 0.83)	0.63	0.82
PC (16:0|18:2)	0.77 (0.72, 0.83)	0.54	0.90
PC (16:0e|22:6)	0.77 (0.72, 0.83)	0.66	0.83
PC (18:2p|20:3)	0.77 (0.71, 0.82)	0.61	0.83
PC (18:2p|18:0)	0.76 (0.70, 0.81)	0.68	0.72
PC (18:0|18:2)	0.74 (0.69, 0.80)	0.61	0.79
PC (16:0|20:4)	0.73 (0.67, 0.79)	0.56	0.88
LPC 22:6	0.73 (0.67, 0.78)	0.55	0.88
LPC 20:4	0.72 (0.66, 0.77)	0.53	0.86
PC (16:0|18:1)	0.71 (0.65, 0.77)	0.72	0.63
PC (16:0|16:0)	0.71 (0.65, 0.77)	0.66	0.70
SM (d18:1|18:2)	0.71 (0.65, 0.76)	0.60	0.76
LPC 20:3	0.70 (0.64, 0.76)	0.54	0.84
PC (18:0e|18:0)	0.68 (0.62, 0.74)	0.52	0.80

The diagnostic performance of individual metabolites and a combined panel is summarized.

Br, Boruta; FC, |log_2_ fold change| ≥ 1.5; LPC, lysophosphatidyl choline; PC, phosphatidylcholine; RF, random forest; SM, sphingomyelin.

### Kidney function decline is associated with higher urinary concentrations of lipid metabolites

**Figure 4:**
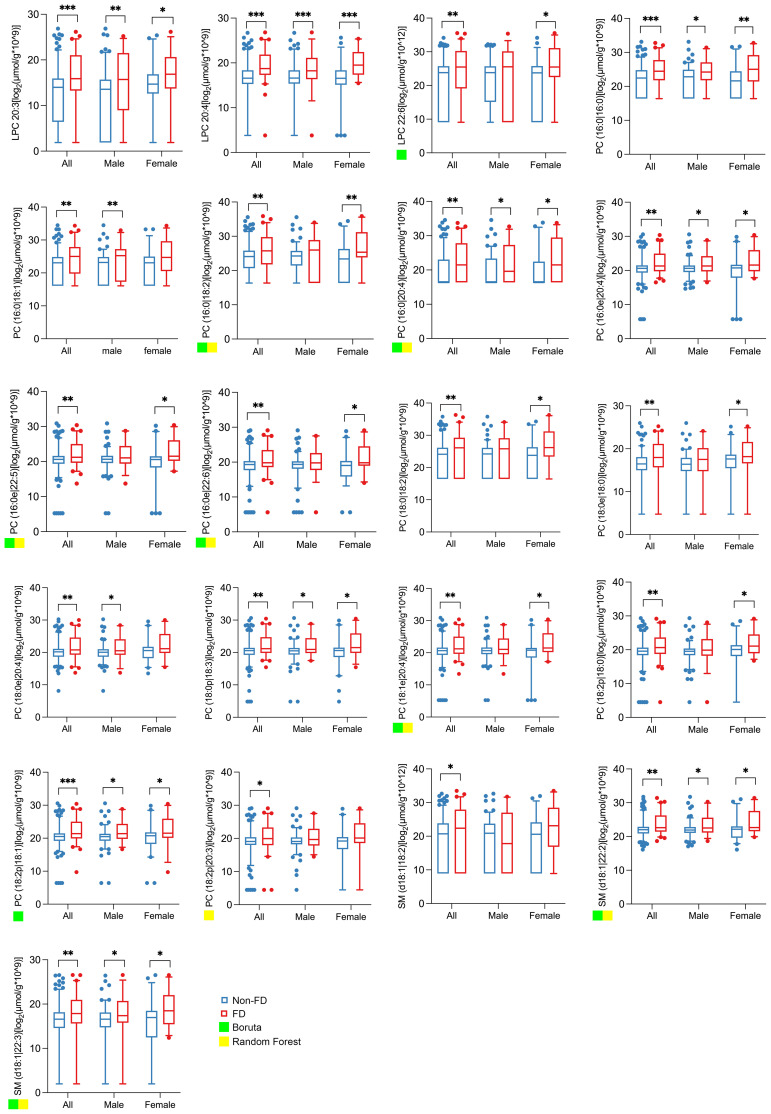
Abundance of the 21 lipid metabolites in spot urine of all, male, and female subjects in future fast decline (FD) and non-FD groups. The boxplots show the abundance of 21 lipid metabolites in the spot urine of all (*n* = 248), male (*n* = 163), and female (*n* = 85) subjects in future FD (*n* = 62) and non-FD (*n* = 186) groups. All lipid concentrations were normalized to urinary creatinine to account for variations in urine concentration. The abundance data were log_2_-transformed prior to statistical analysis and visualization to better approximate a normal distribution. The fast decline (FD) group was defined as the highest quartile of annual eGFR loss. Significance levels are marked as *p < 0.05; **p < 0.01; ***p < 0.001. A green or yellow square indicates that the lipid metabolite is one of the top 10 important differential metabolites selected by Boruta or random forest.

To assess the relationship between urinary lipid metabolites and subsequent kidney function decline, we analyzed a longitudinal cohort of 248 patients with T2D (163 male and 85 female). Based on the quartiles of the annual eGFR decline rate over a median follow-up of 33 months (IQR 17–47), the participants were categorized into a fast decline (FD) group (*n* = 62), with a median eGFR slope of –10.92 mL/min/1.73 m²/year (IQR –18.81 to –6.78), and a non-FD group (*n* = 186), with a median eGFR slope of –1.14 mL/min/1.73 m²/year (IQR –2.64 to 1.23). At baseline, the FD group exhibited higher urinary albumin-to-creatinine ratio (UACR) and a greater proportion of females, though the sex difference was not statistically significant; baseline eGFR was comparable between the two groups (**Table 3**). Notably, the FD group showed significantly higher baseline concentrations of all 21 individual lipid metabolites compared with the non-FD group (**Figure 4**).

**Table 3:** Baseline clinical characteristics of T2D subjects in future fast decline (FD) and Non-FD groups in the longitudinal study.

Clinical characteristics	Non-FD (*n* = 186)	FD (*n* = 62)	*p*-value
Male sex (%)	127 (68.28%)	36 (58.06%)	0.142
Age (year)	57.00 (51.00, 61.25)	58.00 (49.00, 62.25)	0.66
Duration of diabetes (month)	78.00 (13.75, 179.25)	67.00 (8.50, 159.50)	0.561
BMI (kg/m^2^)	25.00 (22.89, 27.47)	24.77 (23.01, 26.69)	0.595
WHR	0.93 ± 0.06	0.95 ± 0.07	0.254
SBP (mmHg)	133.36 ± 16.07	134.93 ± 18.95	0.526
DBP (mmHg)	84.00 (75.00, 90.00)	80.00 (74.00, 90.00)	0.223
HR (per min)	78.00 (71.00, 85.00)	78.00 (70.50, 85.00)	0.886
Hb (g/L)	143.36 ± 17.08	136.52 ± 19.22	0.012
Alb (g/L)	45.10 (42.40, 48.80)	41.80 (37.43, 46.10)	<0.001
FBG (mmol/L)	8.35 (6.92, 11.96)	9.50 (6.47, 11.96)	0.349
HbA1c (%)	7.90 (6.65, 9.60)	9.20 (7.40, 10.80)	0.001
SCr (μmol/L)	69.35 (57.93, 87.63)	69.25 (55.55, 86.58)	0.817
UA (μmol/L)	324.00 (264.75, 384.25)	304.00 (257.00, 380.00)	0.442
TC (mmol/L)	4.51 (3.77, 5.19)	4.95 (4.25, 6.12)	0.004
TG (mmol/L)	1.61 (1.06, 2.56)	1.73 (1.18, 2.44)	0.666
HDL-C (mmol/L)	1.09 (0.94, 1.29)	1.10 (0.96, 1.32)	0.566
LDL-C (mmol/L)	2.95 ± 1.14	3.47 ± 1.36	0.004
UACR (mg/g)	23.10 (7.30, 102.41)	54.54 (14.78, 1,424.86)	<0.001
eGFR (mL/min/1.73^2^)	98.01 (80.01, 106.65)	95.74 (74.77, 108.81)	0.940

Data are presented as mean ± SD, median (IQR), or *n* (%).

Alb, albumin; BMI, body mass index; DBP, diastolic blood pressure; eGFR, estimated glomerular filtration rate; FBG, fast blood glucose; Hb, hemoglobin; HbA1c, hemoglobin A1c; HDL-C, high density lipoprotein cholesterol; HR, heart rate; LDL-C, low density lipoprotein cholesterol; FD, fast decline; SBP, systolic blood pressure; SCr, serum creatinine; TC, total cholesterol; TG, triglyceride; UA, uric acid; UACR, urinary albumin creatinine ratio; WHR, waist–hip ratio.

To evaluate the independent and incremental predictive value of the urinary lipid signature, we performed multivariate logistic regression. After adjusting for established clinical risk factors, including age, sex, baseline eGFR, HbA1c, and albuminuria, the 21-lipid metabolite panel, the eight- and nine-lipid metabolite panels, and LPC 20:4 each remained significant independent predictors of rapid kidney function decline (**Supplementary Table S2**).

### Changes in urinary lipid metabolites are associated with the fast decline in kidney function in individuals with T2D

The predictive performance was further compared using ROC curves (**Table 4**). The ROC curve analysis indicated that the combined panel of all 21 lipid metabolites achieved the highest predictive accuracy for rapid kidney function decline, with an AUC of 0.80. Among individual metabolites, LPC 20:4 demonstrated the strongest predictive value (AUC = 0.72). The eight-metabolite panel derived from RF analysis yielded an AUC of 0.70, with a specificity of 0.83, outperforming both the full 21-metabolite panel and LPC 20:4 in specificity. The predictive model based on clinical variables (including baseline eGFR, HbA1c, and albuminuria) alone achieved an AUC of 0.70. The addition of the 21-lipid metabolite panel significantly improved the predictive performance, yielding a combined AUC of 0.79 (*p* for comparison = 0.002). However, this combined AUC did not exceed that of the 21-metabolite lipid panel alone, indicating that the predictive power of the combined model is predominantly driven by the lipid panel, with clinical variables contributing limited additional information.

**Table 4:** ROC analyses of lipid metabolites and clinical variables for the fast decline of renal function in patients with diabetes in a 33-month longitudinal study.

Panel of lipid metabolites and clinical variables	AUC (95% CI)	Sensitivity	Specificity
FC (*n* = 21)	0.80 (0.74, 0.86)	0.77	0.70
FC + CV (*n* = 24)	0.79 (0.73, 0.86)	0.61	0.86
FC + RF + CV (*n* = 11)	0.74 (0.66, 0.81)	0.54	0.84
FC + Br + CV (*n* = 12)	0.74 (0.66, 0.81)	0.63	0.76
LPC 20:4	0.72 (0.65, 0.79)	0.79	0.58
CV (*n* = 3)	0.70 (0.62, 0.78)	0.70	0.63
FC + RF (*n* = 8)	0.70 (0.62, 0.77)	0.50	0.83
FC + Br (*n* = 9)	0.67 (0.59, 0.75)	0.48	0.80
LPC 20:3	0.67 (0.59, 0.75)	0.66	0.69
PC (16:0|16:0)	0.66 (0.58, 0.74)	0.44	0.82
PC (18:2p|18:1)	0.64 (0.56, 0.73)	0.39	0.91
SM (d18:1|22:2)	0.64 (0.55, 0.72)	0.42	0.90
PC (16:0|18:1)	0.64 (0.55, 0.72)	0.52	0.77
SM (d18:1|22:3)	0.64 (0.55, 0.72)	0.44	0.89
PC (18:0p|18:3)	0.64 (0.55, 0.72)	0.45	0.86
PC (16:0e|20:4)	0.63 (0.55, 0.72)	0.39	0.91
PC (18:2p|18:0)	0.63 (0.54, 0.71)	0.45	0.82
PC (16:0e|22:6)	0.63 (0.54, 0.71)	0.37	0.90
PC (18:0e|18:0)	0.62 (0.54, 0.71)	0.32	0.94
PC (18:0e|20:4)	0.63 (0.54, 0.71)	0.39	0.90
PC (18:1e|20:4)	0.62 (0.54, 0.71)	0.39	0.91
PC (16:0|18:2)	0.62 (0.53, 0.71)	0.39	0.90
PC (18:0|18:2)	0.62 (0.53, 0.71)	0.40	0.87
PC (16:0e|22:5)	0.62 (0.53, 0.71)	0.39	0.91
LPC 22:6	0.62 (0.53, 0.70)	0.55	0.71
PC (16:0|20:4)	0.61 (0.52, 0.70)	0.37	0.91
PC (18:2p|20:3)	0.60 (0.51, 0.69)	0.39	0.91
SM (d18:1|18:2)	0.59 (0.50, 0.68)	0.39	0.89

The predictive performance of the clinical, lipid, and combined models for forecasting rapid renal function decline is summarized.

Br, Boruta; CV, clinical variables, including baseline eGFR, HbA1c and UACR; FC, |log_2_ fold change| ≥ 1.5; LPC, lysophosphatidyl choline; PC, phosphatidylcholine; RF, random forest; SM, sphingomyelin.

We have now performed additional analyses using the common thresholds: a fixed eGFR decline of –5 or –10 mL/min/1.73 m²/year or a 40% decline in eGFR from baseline. The results of these analyses are now presented in **Supplementary Tables S3**–**S5**, respectively. We are pleased to report that the predictive performance of our urinary lipid metabolite panel remains robust and statistically significant across all of these alternative definitions of rapid progression. This consistency greatly strengthens our conclusion that urinary lipids are reliable predictors of future kidney function decline in patients with T2D irrespective of the specific threshold used.

## Discussion

Our lipidomic analysis revealed a distinct urinary lipid profile in patients with DKD, characterized by significant alterations in major lipid classes and elevated levels of specific metabolites. Furthermore, we established that higher baseline concentrations of these differential lipid metabolites are implicated in the future decline of kidney function among individuals with type 2 diabetes, highlighting their potential as prognostic biomarkers for disease progression.

A key advantage of utilizing urinary lipidomics in DKD, as opposed to conventional plasma-based approaches, lies in its potential to offer a more direct and kidney-specific readout of pathological processes. While plasma lipidomics provides a systemic overview, it can be confounded by non-renal metabolic alterations inherent to diabetes. In contrast, the lipid species detected in urine are likely derived from several pathophysiologically relevant sources: they may originate from the shedding of lipid-laden tubular cells, reflect the breakdown of the apical membrane under lipotoxic stress, or directly leak from damaged podocytes. This intra-renal origin positions urinary lipids as a rich source of biomarkers that intimately mirror the local tissue injury and metabolic perturbations within the kidney. While we acknowledge that urine is inevitably influenced by the circulating lipidome, we posit that the disease-specific alterations in this “contaminated” matrix are precisely the signals we aim to capture. Notably, from a clinical translation perspective, urine is a completely non-invasive and readily available biofluid, making any discovered biomarkers more applicable for routine screening and monitoring. Consequently, urinary lipid profiling not only serves as a sensitive prognostic tool but also provides invaluable pathophysiological insights into the progression of DKD, paving the way for future studies.

Our analysis yields a critical insight. The superior predictive capacity of the lipid panel, to which clinical variables added minimal incremental value, strongly suggests that it captures the core pathophysiological signal of rapid renal decline. It is plausible that these urinary lipids directly reflect key disease-specific processes, such as tubular injury, oxidative stress, or specific inflammatory pathways, that are not fully captured by conventional clinical metrics. From a translational perspective, this finding indicates that a more streamlined, lipid-based prognostic tool could offer a highly accurate and potentially more cost-effective strategy to identify high-risk patients.

However, the observation that the combined model did not outperform the lipid-only model also raises important questions. The diminished marginal contribution of clinical variables may point to underlying collinearity, where factors like hyperglycemia and dyslipidemia exert their influence precisely by altering the lipid metabolism represented in our signature. Therefore, while our results underscore the paramount importance of the lipid signature, future studies in larger, independent cohorts are warranted to confirm its standalone power and to disentangle the complex interplay between conventional risk factors and the underlying lipid biology in DKD progression.

The robustness of our urinary lipid metabolite signature was further substantiated through sensitivity analyses employing alternative, standardized definitions of rapid kidney function decline. Crucially, the predictive performance remained statistically significant across all of these thresholds. This consistency underscores that the association is not contingent on a single, arbitrarily chosen cutoff point, thereby strengthening the broader clinical applicability of our findings. While the primary analysis utilized a study-specific quartile to maximize statistical power, these supplementary analyses confirm that the biomarker signature is a reliable predictor of renal function decline, independent of the specific progression criterion applied.

Quantitative data for the prediction of progression of kidney disease in patients with T2D are scarce. Although albuminuria and eGFR are the most important factors, their predictive abilities are modest, including clinical or laboratory parameters that barely improve (23). Abnormal metabolism and intrarenal accumulation of lipids, including phospholipids (13, 24, 25), sphingolipids (26, 27), cholesterol (28), triglycerides, and fatty acids (29–31), are strongly associated with the development and progression of DKD. A meta-analysis of 34 clinical-based metabolomics studies identified five essential metabolites related to DKD compared with healthy control, suggesting lipid metabolism pathways related to DKD (32).

Modern lipidomics based on mass spectrometry have enabled the identification and quantitation of lipids (33). Patients with DKD had higher plasma/serum acylcarnitines compared with diabetic patients without a kidney disease (34–36), which also predicted DKD progression (34, 36–38). Lower long-chain acylcarnitine and higher medium- and short-chain acylcarnitine were associated with GFR reduction in patients with DKD (39). Double-bond TAG species and polyunsaturated PE species were predictors of DKD progression in T2D (40). Circulating ceramide (Cer) (18:1/16:0) and Cer(18:1/16:1) were significantly increased in patients with T2D DKD compared with patients with T2D without DKD (35), while very long chain (C20–26) ceramides were significantly decreased in T1DM DKD patients as compared with T1DM without albuminuria (41). Serum integrative omics of proteomes and metabolomes revealed serum glycerol-3-galactoside as an independent marker to predict DKD (42). A previous genome-wide association study and a subsequent two-sample Mendelian randomization analysis revealed that urinary ethanolamine, an initial precursor for PC, was associated with higher eGFR in people with T1D (43). These findings establish targeted lipidomics as a feasible and reliable approach to evaluate kidney damage in diabetes.

We found that SM (d18:1|22:3) and SM (d18:1|22:2) had identical and the highest AUC of 0.80 among the 21 individual lipid metabolites. SM was a significant biochemical covariate of urine albumin and the strongest lipid regressor for kidney disease in human T1D (44). Serum SM was also highly upregulated in DKD and significantly correlated to albuminuria (45). Lower serum SM species was reported in participants with macroalbuminuria compared with those with normo-albuminuria, while SM (d18:1|24:0), SM (d40:1), and SM (d41:1) were associated with a lower risk of kidney disease progression or mortality (46). Here PC species constituted the predominant fraction of the differential lipid metabolites. High-fat-diet-induced upregulation of PC-enriched exosome contributed to insulin resistance (47). LPC was dramatically elevated in the early stage of DKD (48). We showed that LPC 20:4 was a strong predictor of FD in the kidney function of T2D. Coincidentally, urinary LPC (16:0) and LPC (18:0) increased in the fast decliner of stage G3 DKD. Moreover, tubular accumulation of LPC enhanced organelle stress and cell apoptosis, accelerating tubular lipotoxicity (13). These results provided evidence that specific lipid metabolite biomarkers also play critical roles in regulating the pathogenesis of DKD.

Our findings both corroborate and extend previous knowledge. In line with prior research, we confirm that lipid metabolism is profoundly disturbed in DKD, observing elevated levels of specific lipid classes previously implicated in diabetic complications. More importantly, our study provides several novel insights. First, we identify a specific urinary lipid signature that is not merely associated with the presence of DKD but is predictive of its future rate of progression. This prognostic capacity, validated in a longitudinal cohort, addresses a critical gap in the field. Second, we demonstrate that this urinary lipidomic profile provides significant independent and incremental prognostic information over standard clinical markers, including albuminuria, glucose, and eGFR, and underscores its potential clinical utility to identifying high-risk patients who might be missed by current metrics. Finally, by focusing on urine, we highlight a direct, kidney-related lipidomic footprint that is more accessible for clinical translation than plasma or biopsy-based markers.

We acknowledge several limitations. The longitudinal findings require confirmation in larger, multi-center cohorts to ensure robustness. Furthermore, the observational nature of our study cannot establish causality; mechanistic investigations are essential to determine if these lipid metabolites are indeed drivers of pathology. We acknowledge that intra-individual variation exists for any biomarker. However, by studying a sizable cohort and employing rigorous normalization, we are confident that the strong associations and predictive signals we observed robustly reflect the underlying biology. The absence of detailed medication information (e.g., on adherence and specific dosages) represents a limitation, as it precludes a comprehensive analysis of how pharmacotherapy might interact with or influence the urinary lipidome. Future prospective studies designed to incorporate detailed drug monitoring are warranted to validate our findings and to explore the interplay between medications and lipid metabolism in DKD. Finally, it is important to consider that our study lacked a healthy control cohort. Therefore, the lipidomic differences identified here specifically reflect changes associated with DKD progression within a diabetic population but do not define the broader shifts that occur from a state of health to diabetes. Establishing this “healthy baseline” in future work will be crucial for a complete understanding of the pathogenic timeline of lipid dysregulation in DKD.

In conclusion, our study establishes specific urinary lipid metabolites as non-invasive biomarkers that not only reflect but also predict the progression of DKD. By demonstrating their incremental value over standard clinical parameters, this work provides a foundation for improved risk stratification. Ultimately, the dysregulated lipids identified here offer two pivotal opportunities: first, as a source of mechanistic insights for future research, and second, as a foundation to develop novel prognostic tools and targeted therapies to alter the trajectory of kidney disease in T2D.

## Data Availability

The raw data supporting the conclusions of this article will be made available by the authors, without undue reservation.
