# Supplementary material for: Urinary lipid metabolites and progression of kidney disease in individuals with type 2 diabetes
Source: Front Endocrinol (Lausanne). 2025 Nov 19;16:1650498. doi: 10.3389/fendo.2025.1650498 (PMC12672268; doi:10.3389/fendo.2025.1650498)
Supplement: Supplementary file 1 [file DataSheet1.pdf]

Supplementary material for

Urinary lipid metabolites and progression of kidney disease in individuals with type 2 diabetes

Yu Xiao, Caifeng Shi, Songyan Qin, Aiqin He, Xiaomei Wu, Chunsun Dai, Yang Zhou

File list

Supplementary Table 1 Multivariable linear regression of lipid metabolites on DKD status.

Supplementary Table 2 Multivariable logistic regression analyses for predicting rapid kidney function decline.

Supplementary Table 3 ROC analyses of lipid metabolites for predicting fast decline of renal function (defined as an eGFR slope of < -5 mL/min/1.73 m^2/year) in patients with diabetes in a 33-month longitudinal study (n = 65).

Supplementary Table 4 ROC analyses of lipid metabolites for predicting fast decline of renal function (defined as an eGFR slope of < -10 mL/min/1.73 m^2/year) in patients with diabetes in a 33-month longitudinal study (n = 34).

Supplementary Table 5 ROC analyses of lipid metabolites for predicting fast decline of renal function (defined as eGFR loss as 40% decline from baseline) in patients with diabetes in a 33-month longitudinal study (n = 19).

Supplementary Fig. 1 Comprehensive volcano plot of lipidomic changes.

Supplementary Fig. 2 Correlation analysis of the identified lipid metabolites.

Supplementary Table 1 Multivariable linear regression of lipid metabolites on DKD status.

	B	OR	95%CI	*p* value
LPC 20:3				
Crude Model	0.339	1.403	(1.241, 1.585)	<0.001
Adjust Model	0.355	1.426	(1.249, 1.628)	<0.001
LPC 20:4				
Crude Model	0.662	1.939	(1.508, 2.493)	<0.001
Adjust Model	0.689	1.993	(1.515, 2.621)	<0.001
LPC 22:6				
Crude Model	0.312	1.367	(1.224, 1.526)	<0.001
Adjust Model	0.311	1.365	(1.216, 1.533)	<0.001
PC(16:0e|20:4)				
Crude Model	1.463	4.320	(2.736, 6.821)	<0.001
Adjust Model	1.435	4.198	(2.630, 6.700)	<0.001
PC(16:0e|22:5)				
Crude Model	1.447	4.251	(2.701, 6.690)	<0.001
Adjust Model	1.447	4.252	(2.656, 6.808)	<0.001
PC(16:0e|22:6)				
Crude Model	0.814	2.256	(1.680, 3.030)	<0.001
Adjust Model	0.775	2.171	(1.610, 2.928)	<0.001
PC(18:0e|18:0)				
Crude Model	0.461	1.585	(1.304, 1.927)	<0.001
Adjust Model	0.505	1.657	(1.338, 2.052)	<0.001
PC(18:0e|20:4)				
Crude Model	1.588	4.893	(3.055, 7.835)	<0.001
Adjust Model	1.535	4.641	(2.881, 7.476)	<0.001
PC(18:0p|18:3)				
Crude Model	1.498	4.471	(2.787, 7.174)	<0.001
Adjust Model	1.470	4.351	(2.678, 7.068)	<0.001
PC(18:1e|20:4)				
Crude Model	1.389	4.009	(2.583, 6.223)	<0.001

Adjust Model	1.385	3.995	(2.535, 6.296)	<0.001
PC(18:2p|18:0)				
Crude Model	1.253	3.502	(2.319, 5.288)	<0.001
Adjust Model	1.296	3.656	(2.358, 5.667)	<0.001
PC(18:2p|18:1)				
Crude Model	1.442	4.229	(2.687, 6.658)	<0.001
Adjust Model	1.447	4.251	(2.652, 6.813)	<0.001
PC(18:2p|20:3)				
Crude Model	0.885	2.423	(1.754, 3.346)	<0.001
Adjust Model	0.880	2.412	(1.731, 3.362)	<0.001
PC(16:0|16:0)				
Crude Model	0.580	1.786	(1.483, 2.150)	<0.001
Adjust Model	0.612	1.844	(1.512, 2.250)	<0.001
PC(16:0|18:1)				
Crude Model	0.549	1.731	(1.444, 2.074)	<0.001
Adjust Model	0.574	1.776	(1.465, 2.153)	<0.001
PC(16:0|18:2)				
Crude Model	0.763	2.144	(1.735, 2.650)	<0.001
Adjust Model	0.797	2.219	(1.766, 2.789)	<0.001
PC(16:0|20:4)				
Crude Model	0.733	2.081	(1.698, 2.550)	<0.001
Adjust Model	0.793	2.209	(1.769, 2.759)	<0.001
PC(18:0|18:2)				
Crude Model	0.584	1.793	(1.505, 2.135)	<0.001
Adjust Model	0.607	1.835	(1.521, 2.215)	<0.001
SM(d18:1|18:2)				
Crude Model	0.315	1.371	(1.231, 1.526)	<0.001
Adjust Model	0.321	1.379	(1.229, 1.547)	<0.001
SM(d18:1|22:2)				
Crude Model	2.044	7.725	(4.270, 13.977)	<0.001
Adjust Model	2.024	7.570	(4.070, 14.080)	<0.001
SM(d18:1|22:3)				

Crude Model	0.736	2.089	(1.636, 2.667)	<0.001
Adjust Model	0.740	2.095	(1.625, 2.702)	<0.001

The table presents the results of separate multivariable linear regression models for each of the 21 lipid metabolites. All models were adjusted for potential clinical confounders, including diabetes duration, HbA1c, and serum lipid profiles. The dependent variable was the lipid metabolite level, and the independent variable of interest was the DKD status. A positive regression coefficient (B) indicates a higher level of the lipid in the DKD group relative to the uncomplicated diabetes group, after accounting for the covariates. LPC, lysophosphatidyl choline; PC, phosphatidylcholine; RF, random forest; SM, sphingomyelin.

Supplementary Table 2 Multivariable logistic regression analyses for predicting rapid kidney function decline.

	B	OR	95%CI	*p* value
FC (n = 21)				
Crude Model	5.12	166.65	(33.40, 831.49)	<0.001
Adjust Model	5.34	209.33	(23.89, 1834.62)	<0.001
FC + RF (n = 8)				
Crude Model	4.83	125.24	(16.89, 928.52)	<0.001
Adjust Model	5.99	397.73	(7.87, 20098.30)	0.003
FC + Br (n = 9)				
Crude Model	4.70	109.87	(13.19, 914.97)	<0.001
Adjust Model	6.23	507.25	(5.84, 44031.18)	0.006
LPC 20:3				
Crude Model	0.27	1.31	(1.12, 1.53)	<0.001
Adjust Model	0.18	1.20	(0.99, 1.45)	0.059
LPC 20:4				
Crude Model	0.71	2.03	(1.48, 2.78)	<0.001
Adjust Model	0.58	1.79	(1.16, 2.76)	0.008
LPC 22:6				
Crude Model	0.15	1.16	(1.02, 1.33)	0.026
Adjust Model	0.06	1.06	(0.90, 1.25)	0.485
PC(16:0e|20:4)				
Crude Model	0.65	1.91	(1.38, 2.64)	<0.001
Adjust Model	0.52	1.68	(0.93, 3.05)	0.087
PC(16:0e|22:5)				
Crude Model	0.55	1.74	(1.28, 2.37)	<0.001
Adjust Model	0.29	1.34	(0.80, 2.23)	0.264
PC(16:0e|22:6)				
Crude Model	0.49	1.63	(1.22, 2.18)	<0.001
Adjust Model	0.22	1.25	(0.81, 1.92)	0.312
PC(18:0e|18:0)				

Crude Model	0.22	1.24	(0.99, 1.56)	0.061
Adjust Model	-0.10	0.90	(0.70, 1.17)	0.438
PC(18:0e|20:4)				
Crude Model	0.58	1.78	(1.30, 2.43)	<0.001
Adjust Model	0.37	1.44	(0.81, 2.56)	0.210
PC(18:0p|18:3)				
Crude Model	0.66	1.93	(1.40, 2.68)	<0.001
Adjust Model	0.54	1.71	(0.95, 3.08)	0.072
PC(18:1e|20:4)				
Crude Model	0.56	1.75	(1.29, 2.39)	<0.001
Adjust Model	0.33	1.39	(0.83, 2.34)	0.209
PC(18:2p|18:0)				
Crude Model	0.51	1.66	(1.22, 2.26)	0.001
Adjust Model	0.20	1.22	(0.81, 1.85)	0.340
PC(18:2p|18:1)				
Crude Model	0.60	1.82	(1.32, 2.50)	<0.001
Adjust Model	0.37	1.45	(0.85, 2.46)	0.174
PC(18:2p|20:3)				
Crude Model	0.36	1.43	(1.08, 1.89)	0.012
Adjust Model	0.10	1.11	(0.76, 1.62)	0.605
PC(16:0|16:0)				
Crude Model	0.43	1.54	(1.24, 1.93)	<0.001
Adjust Model	0.24	1.27	(0.94, 1.71)	0.115
PC(16:0|18:1)				
Crude Model	0.31	1.37	(1.11, 1.69)	0.004
Adjust Model	0.16	1.17	(0.88, 1.57)	0.286
PC(16:0|18:2)				
Crude Model	0.30	1.35	(1.10, 1.66)	0.004
Adjust Model	0.06	1.06	(0.79, 1.42)	0.693
PC(16:0|20:4)				
Crude Model	0.31	1.36	(1.13, 1.63)	0.001
Adjust Model	0.08	1.09	(0.80, 1.47)	0.590

PC(18:0|18:2)				
Crude Model	0.26	1.29	(1.07, 1.56)	0.008
Adjust Model	0.04	1.04	(0.80, 1.36)	0.763
SM(d18:1|18:2)				
Crude Model	0.12	1.13	(1.00, 1.28)	0.056
Adjust Model	-0.05	0.95	(0.81, 1.13)	0.576
SM(d18:1|22:2)				
Crude Model	0.74	2.09	(1.48, 2.95)	<0.001
Adjust Model	0.69	1.99	(1.00, 3.95)	0.050
SM(d18:1|22:3)				
Crude Model	0.32	1.37	(1.10, 1.72)	0.005
Adjust Model	0.10	1.11	(0.86, 1.43)	0.438

The table presents the results of separate multivariable logistic regression models for the combined lipid panel and for each of the 21 individual lipid metabolites. All models were adjusted for potential clinical confounders, including age, sex, baseline eGFR, HbA1c, and albuminuria. The outcome was belonging to the fast decline group. An OR > 1 indicates that a higher level of the lipid is associated with increased odds of being in the fast decline group. LPC, lysophosphatidyl choline; PC, phosphatidylcholine; RF, random forest; SM, sphingomyelin.

Supplementary Table 3 ROC analyses of lipid metabolites for predicting fast decline of renal function (defined as an eGFR slope of < -5 mL/min/1.73 m²/year) in patients with diabetes in a 33-month longitudinal study (n = 65).

	AUC (95%CI)	Sensitivity	Specificity
FC (n = 21)	0.76 (0.69, 0.83)	0.74	0.70
LPC 20:4	0.69 (0.62, 0.77)	0.75	0.57
FC + RF (n = 8)	0.67 (0.59, 0.75)	0.48	0.83
FC + Br (n = 9)	0.65 (0.57, 0.74)	0.34	0.92
LPC 20:3	0.64 (0.55, 0.72)	0.63	0.69
PC (16:0|16:0)	0.63 (0.55, 0.72)	0.39	0.85
PC (16:0|18:1)	0.62 (0.53, 0.71)	0.51	0.78
PC (18:2p|18:1)	0.61 (0.52, 0.70)	0.37	0.91
SM (d18:1|22:2)	0.61 (0.52, 0.70)	0.40	0.90
PC (18:0|18:2)	0.61 (0.52, 0.69)	0.39	0.87
PC (18:0p|18:3)	0.61 (0.52, 0.69)	0.42	0.87
SM (d18:1|22:3)	0.60 (0.52, 0.69)	0.42	0.89
PC (18:2p|18:0)	0.60 (0.52, 0.69)	0.43	0.82
PC (16:0|18:2)	0.60 (0.51, 0.69)	0.37	0.90
PC (16:0e|20:4)	0.60 (0.51, 0.69)	0.37	0.91
PC (18:0e|18:0)	0.60 (0.51, 0.69)	0.31	0.93
PC (16:0|20:4)	0.60 (0.51, 0.69)	0.35	0.91
PC (16:0e|22:6)	0.60 (0.51, 0.68)	0.35	0.90
LPC 22:6	0.60 (0.51, 0.68)	0.39	0.85
PC (18:0e|20:4)	0.60 (0.51, 0.68)	0.37	0.89
PC (18:1e|20:4)	0.59 (0.51, 0.68)	0.37	0.91
PC (16:0e|22:5)	0.59 (0.50, 0.68)	0.37	0.91
PC (18:2p|20:3)	0.57 (0.48, 0.66)	0.37	0.91
SM (d18:1|18:2)	0.57 (0.48, 0.66)	0.37	0.89

The predictive performance of the lipid metabolite models for forecasting rapid renal function decline is summarized. Br, Boruta; FC, |log2 fold change|≥1.5; LPC, lysophosphatidyl choline; PC, phosphatidylcholine; RF, random forest; SM, sphingomyelin.

Supplementary Table 4 ROC analyses of lipid metabolites for predicting fast decline of renal function (defined as an eGFR slope of < -10 mL/min/1.73 m²/year) in patients with diabetes in a 33-month longitudinal study (n = 34).

	AUC (95%CI)	Sensitivity	Specificity
FC (n = 21)	0.81 (0.75, 0.88)	0.85	0.65
LPC 20:4	0.79 (0.71, 0.86)	0.74	0.74
FC + RF (n = 8)	0.77 (0.68, 0.86)	0.62	0.89
FC + Br (n = 9)	0.76 (0.66, 0.85)	0.56	0.90
PC (18:0p|18:3)	0.74 (0.64, 0.84)	0.59	0.88
SM (d18:1|22:2)	0.73 (0.63, 0.83)	0.59	0.89
PC (18:2p|18:1)	0.73 (0.63, 0.83)	0.59	0.89
SM (d18:1|22:3)	0.73 (0.62, 0.83)	0.65	0.86
PC (18:1e|20:4)	0.72 (0.62, 0.83)	0.56	0.90
PC (16:0|18:2)	0.72 (0.61, 0.83)	0.59	0.90
PC (16:0e|20:4)	0.72 (0.61, 0.82)	0.56	0.90
PC (16:0e|22:5)	0.72 (0.61, 0.82)	0.56	0.90
PC (18:0|18:2)	0.71 (0.61, 0.82)	0.65	0.79
PC (16:0e|22:6)	0.71 (0.60, 0.82)	0.59	0.86
PC (18:2p|18:0)	0.71 (0.60, 0.82)	0.65	0.83
PC (16:0|20:4)	0.71 (0.60, 0.82)	0.62	0.83
PC (18:0e|20:4)	0.71 (0.60, 0.81)	0.56	0.90
PC (16:0|16:0)	0.71 (0.60, 0.81)	0.59	0.81
LPC 20:3	0.70 (0.60, 0.80)	0.74	0.66
PC (18:2p|20:3)	0.70 (0.59, 0.81)	0.59	0.87
PC (16:0|18:1)	0.70 (0.59, 0.81)	0.59	0.86
LPC 22:6	0.69 (0.58, 0.80)	0.71	0.70
PC (18:0e|18:0)	0.69 (0.58, 0.80)	0.47	0.90
SM (d18:1|18:2)	0.65 (0.53, 0.77)	0.56	0.88

The predictive performance of the lipid metabolite models for forecasting rapid renal function decline is summarized., Boruta; FC, \|log2 fold change\|≥1.5; LPC, lysophosphatidyl choline; PC, phosphatidylcholine; RF, random forest; SM, sphingomyelin.

Supplementary Table 5 ROC analyses of lipid metabolites for predicting fast decline of renal function (defined as eGFR loss as 40% decline from baseline) in patients with diabetes in a 33-month longitudinal study (n = 19).

	AUC (95%CI)	Sensitivity	Specificity
PC (16:0|16:0)	0.94 (0.91, 0.97)	1.00	0.85
PC (18:2p|18:0)	0.94 (0.91, 0.98)	0.95	0.88
PC (18:2p|18:1)	0.94 (0.90, 0.98)	0.95	0.92
PC (18:2p|20:3)	0.94 (0.90, 0.98)	0.95	0.91
PC (16:0e|22:5)	0.94 0.89, 0.98)	0.95	0.91
PC (18:0p|18:3)	0.94 (0.89, 0.98)	0.95	0.91
PC (18:1e|20:4)	0.94 (0.89, 0.98)	0.95	0.91
SM (d18:1|22:3)	0.94 (0.90, 0.98)	0.95	0.90
PC (18:0e|20:4)	0.94 (0.89, 0.98)	0.95	0.89
PC (16:0e|22:6)	0.93 (0.89, 0.98)	0.95	0.88
SM (d18:1|22:2)	0.93 (0.88, 0.99)	0.95	0.92
FC + Br (n = 9)	0.93 (0.88, 0.98)	0.90	0.92
PC (18:0|18:2)	0.93 (0.87, 0.98)	0.90	0.89
PC (16:0e|20:4)	0.92 (0.86, 0.99)	0.95	0.90
SM (d18:1|18:2)	0.92 (0.86, 0.98)	0.84	0.90
FC + RF (n = 8)	0.92 (0.85, 0.98)	0.90	0.89
PC (16:0|18:2)	0.91 (0.84, 0.99)	0.84	0.92
PC (18:0e|18:0)	0.91 (0.84, 0.97)	0.90	0.80
LPC 20:4	0.90 (0.85, 0.95)	0.95	0.87
PC (16:0|20:4)	0.88 (0.78, 0.98)	0.84	0.90
LPC 22:6	0.85 (0.74, 0.96)	0.90	0.84
FC (n = 21)	0.85 (0.79, 0.91)	1.00	0.63
PC (16:0|18:1)	0.83 (0.71, 0.95)	0.74	0.89
LPC 20:3	0.81 (0.71, 0.92)	0.90	0.81

The predictive performance of the lipid metabolite models for forecasting rapid renal function decline is summarized. Br, Boruta; FC, |log2 fold change|≥1.5; LPC, lysophosphatidyl choline; PC, phosphatidylcholine; RF, random forest; SM, sphingomyelin.

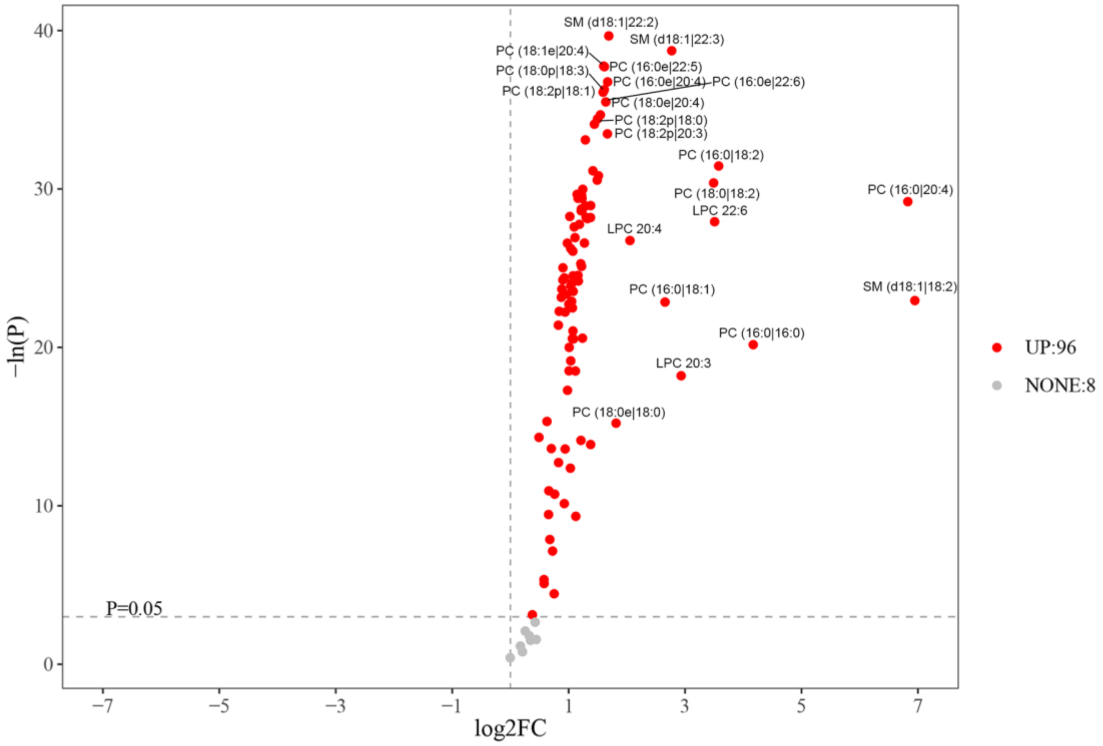

Supplementary Fig. 1 Comprehensive volcano plot of lipidomic changes. This plot displays the fold change against the statistical significance (-ln *p*-value) for all detected lipids (n = 104). The dashed horizontal line indicates the significance threshold ($p < 0.05$). Metabolites above this line and to the right of the vertical line are considered upregulated (highlighted in red, n = 96). The 21 significantly upregulated lipids based on the screening criteria of $|\log_2$ fold change (FC)$| \geq 1.5$ and $p < 0.05$ are specifically labeled.

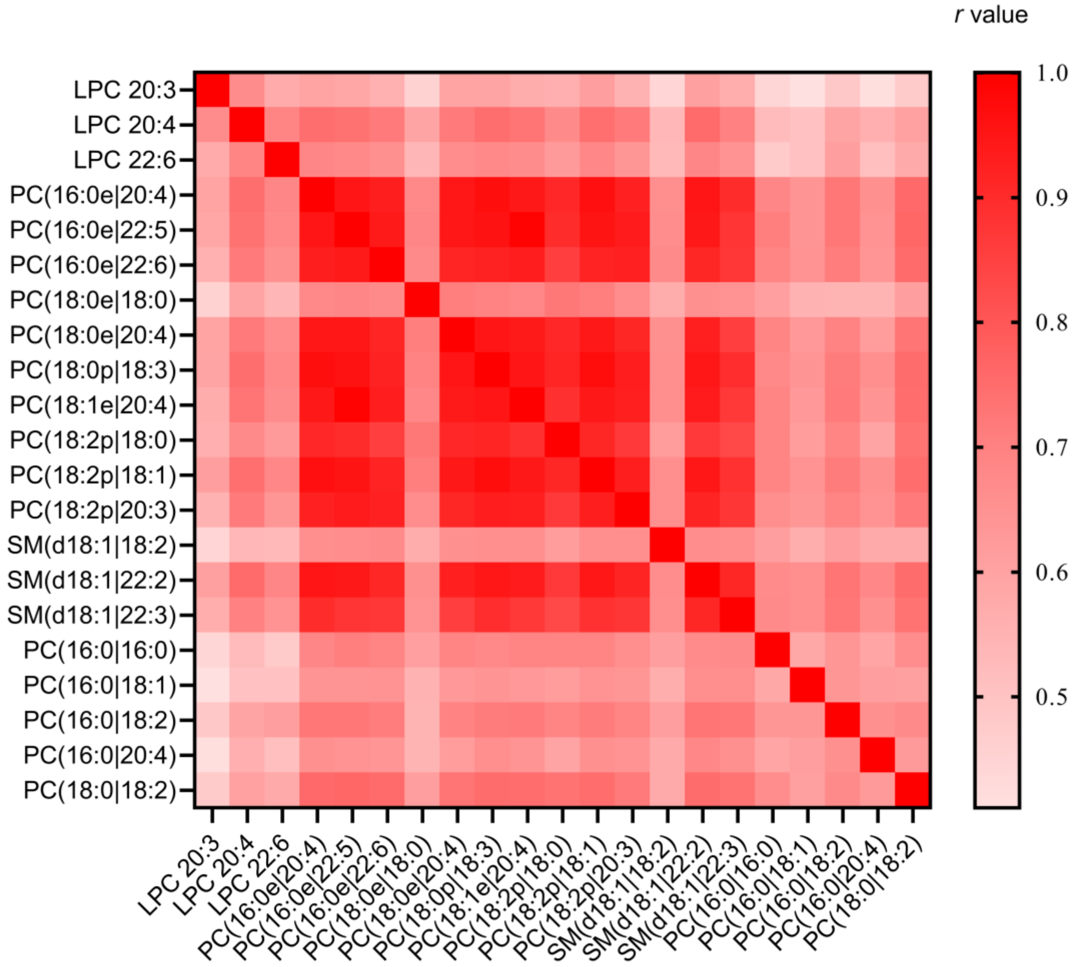

Supplementary Fig. 2 Correlation analysis of the identified lipid metabolites. Pairwise correlation analysis of the lipid metabolites. The heatmap visualizes the correlation coefficients, demonstrating a predominant and high-magnitude positive correlation (red) among the majority of the analyzed lipids, confirming their strong co-regulation.
